# Pharmacokinetics of Orally Administered Phenazopyridine in Goats With Obstructive Urolithiasis

**DOI:** 10.1111/jvim.70167

**Published:** 2025-06-26

**Authors:** Sienna L. Mitman, Danielle A. Mzyk, Blanca E. Camacho, R. McAlister Council‐Troche, Jennifer Davis, Derek M. Foster, Jennifer Halleran

**Affiliations:** ^1^ North Carolina State University, College of Veterinary Medicine Raleigh North Carolina USA; ^2^ North Carolina State University, College of Veterinary Medicine, Department of Population Health and Pathobiology Raleigh North Carolina USA; ^3^ Virginia‐Maryland Regional College of Veterinary Medicine, Department of Biomedical Sciences and Pathobiology Blacksburg Virginia USA

**Keywords:** analgesia, caprine, pharmacology, urolithiasis

## Abstract

**Background:**

Phenazopyridine is used for ancillary pain management in the treatment of goats with obstructive urolithiasis. However, there are no published studies on the pharmacokinetics, safety, or efficacy of phenazopyridine in goats.

**Hypothesis/Objectives:**

Determine the pharmacokinetic parameters of phenazopyridine after oral administration in goats with obstructive urolithiasis after tube cystostomy surgery.

**Animals:**

Six male goats, ages 3 months to 4 years.

**Methods:**

Prospective, observational study. Goats presenting to a veterinary institution diagnosed with obstructive urolithiasis underwent a tube cystostomy surgery. After surgery, phenazopyridine (4 mg/kg PO q12h) was administered. Plasma and urine samples were collected at predetermined intervals, and the concentration of phenazopyridine and clinically relevant metabolites was determined using ultra high‐performance liquid chromatography with mass spectrometry. The pharmacokinetic parameters were determined using non‐compartmental analysis.

**Results:**

The harmonic mean terminal elimination plasma half‐life (*T*_1/2_), geometric mean maximum plasma concentration (*C*_max_), and area under the curve (AUC) were 0.5 h (0.22–1.57 h), 263.4 ng/mL (137.35–1047.88 ng/mL), and 0.69 h*ng/mL (0.10–2.99 h*ng/mL), respectively for phenazopyridine. The concentration of phenazopyridine in urine samples was below the limit of assay detection (1.5 ng/mL) in all but one sample.

**Conclusions and Clinical Importance:**

Phenazopyridine was rapidly eliminated from plasma and did not concentrate at detectable levels in the urine after oral administration.

## 1 Introduction

Obstructive urolithiasis is the most common urinary tract disease of small ruminants [1]. Treatment is dictated by multiple factors such as urolith type and animal use, but surgery (i.e., tube cystostomy) is often warranted [2]. While nonsteroidal anti‐inflammatory medications are indicated upon resolution of azotemia [1], some animals require multimodal analgesic approaches. In these instances, it is common to administer phenazopyridine, a urinary bladder analgesic used commonly in human medicine, but with limited information in small ruminants [3, 4, 5, 6, 7]. In humans, phenazopyridine is commonly used in the treatment of dysuria and has been utilized for a variety of urogenital conditions, including cystitis, prostatitis, and urinary bladder surgery [8, 9, 10].

Brought to market before current regulations, phenazopyridine avoided much of the clinical studies that would be required today [9]. Efficacy, safety, and pharmacokinetic studies in any species, including humans, are therefore limited. Research in humans, rats, and rabbits suggests the pharmacokinetic parameters vary widely [11, 12, 13]. In humans, phenazopyridine is thought to be metabolized in the liver, and as much as 45% is excreted unchanged in the urine, where it is thought to have a local analgesic effect [14]. The precise mechanism of action remains unknown but might be mediated through the inhibition of nociceptive kinases or afferent urinary bladder nerve fibers [14, 15, 16, 17]. Multiple adverse effects, including methemoglobinemia, hepatotoxicity, and acute renal failure, occur with use in humans and small animal species, usually associated with high doses or prolonged administration [18, 19, 20, 21, 22]. These adverse effects are thought to be mediated through metabolites such as aniline and tri‐aminopyridine [14, 23]. The drug is therefore not recommended in humans with renal insufficiency, and seldom used in small animal medicine [19, 23]. In human adults, phenazopyridine is administered at a dose of 100–200 mg by mouth every 8 h, but no clinically effective dose has been established in any non‐human species [9, 19]. Dosing in small ruminants varies from 3 to 5 mg/kg every 8 to 12 h and is largely based on clinician experience [3, 4, 5, 6, 7, 24]. Given the widespread use of phenazopyridine in goats, yet the scarcity of information validating its use in this species, there is a need to establish the pharmacokinetic parameters of orally administered phenazopyridine in goats.

In this study, we thus sought (1) to determine the pharmacokinetics of orally administered phenazopyridine (4 mg/kg every 12 h) and its clinically relevant metabolites (aniline, acetaminophen) in post‐operative goats with obstructive urolithiasis and (2) to use these results to make recommendations regarding the optimal dosing regimen. We hypothesized that the elimination half‐life would be shorter in urine samples, but the maximum concentration would be increased in urine compared to plasma. We believe the derived pharmacokinetic parameters of phenazopyridine would be similar to those reported previously in other species.

## 2 Materials and Methods

### 2.1 Animal Use and Ethics

This study was approved by the North Carolina State University's Institutional Animal Care and Use Committee (PROTOCOL 22‐295). All owners signed an informed consent before enrollment.

### 2.2 Study Cohort and Animal Care

Animals in this study were goats presenting to a veterinary teaching hospital and diagnosed with obstructive urolithiasis. Diagnosis of obstructive urolithiasis was based on history, physical examination, clinicopathologic abnormalities (blood chemistries were performed at admission), and imaging (abdominal ultrasound, radiographs or both). Owners were offered all treatment options after diagnosis. If owners elected to pursue surgical correction via tube cystostomy, and upon owner consent their animal was enrolled in the study.

Before surgery, an intravenous jugular catheter (MILA International Inc., Florence, KY) was placed aseptically in each goat. All goats underwent tube cystostomy surgery in which an indwelling Foley urinary bladder catheter (Bardex Foley Catheter, BARD, Covington GA) was placed under general anesthesia. Uroliths collected at the time of surgery were submitted for analysis to the Minnesota Urolith Center. Goats received any clinically indicated treatments during and after surgery (i.e., intravenous fluids, a nonsteroidal anti‐inflammatory medication, antimicrobials, acepromazine, morphine, and transdermal fentanyl). Goats were housed in individual hospital stalls bedded with shavings. They were fed orchard grass or Timothy hay, or both, ad‐libitum with Timothy pellets provided every 12 h. Goats were offered food as soon as recovered after surgery. Both fresh water and electrolyte water were offered ad‐libitum.

### 2.3 Phenazopyridine Administration

The morning after surgery (time ranged from 4.5 to 14 h after extubation), phenazopyridine (Amerifit Brands, Azoproducts, Cromwell, CT) was administered orally at a dose of 4 mg/kg every 12 h. For administration, the required number of 95 mg phenazopyridine tablets was dissolved in water and administered orally via syringe.

### 2.4 Blood Sampling

**FIGURE 1:**
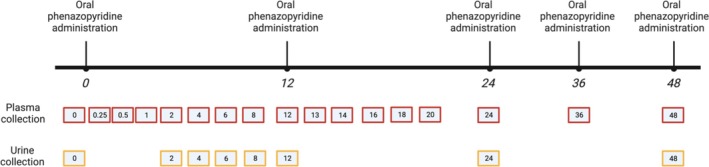
Schematic of phenazopyridine administration and sampling time points for this study. Phenazopyridine was administered orally at a dose of 4 mg/kg every 12 h for 48 h (five total doses). Plasma samples were obtained prior to initial phenazopyridine administration (0 h), and at 0.25, 0.5, 1, 2, 4, 6, 8, 12, 13, 14, 16, 18, 20, 24, 36, and 48 h after initial phenazopyridine administration. Urine samples were obtained prior to initial phenazopyridine administration (0 h), and at 2, 4, 6, 8, 12, 24, 36, and 48 h after initial phenazopyridine administration.

Blood samples (3 mL) were collected from the jugular catheter of study animals at specific time points. A baseline time point 0 sample was collected before phenazopyridine administration and then at 0.25, 0.5, 1, 2, 4, 6, 8, 12, 13, 14, 16, 18, 20, 24, 36, and 48 h after the initial phenazopyridine dose (Figure 1). To collect blood, the intravenous catheter was flushed with dilute heparin and a 12 mL blood sample was collected (Syringe 1). A second syringe (Syringe 2) was then used to collect 3 mL of blood, and this sample was transferred to a lithium heparin blood tube (Becton Dickenson (BD), Franklin Lakes, NJ). The blood from the first syringe (Syringe 1) was then given back to the goat, and the catheter was flushed again with dilute heparin. Blood was placed on ice and transferred, spun at 1000×G for 10 min. Plasma was then separated and stored at −80°C until further analysis.

Although samples were collected after the second and third phenazopyridine doses, sampling intervals were not consistent amongst all enrolled goats beyond the 12‐h timepoint. Therefore, analysis was conducted on blood samples collected 0–12 h after initial dose.

### 2.5 Urine Sampling

Urine samples were collected from the Foley catheter immediately before phenazopyridine administration (time point 0) and at 2, 4, 6, 8, 12, 24, 36, and 48 h after initial phenazopyridine dose (Figure 1). To collect a sample, the Foley catheter was clamped for 5–10 min, then the clamp was removed and voided urine was collected into a tube (50 mL conical tube, Sigma Aldrich) with no additives. Samples were stored at −80°C until further analysis.

### 2.6 Phenazopyridine, Acetaminophen, and Aniline Concentration Determination

Samples were analyzed at the VA‐MD College of Veterinary Medicine Analytical Chemistry Research Laboratory. Analyte concentrations in plasma and urine samples were determined by ultra high‐performance liquid chromatography with tandem mass spectrometry (UPLC–MS/MS). Reference standards of phenazopyridine hydrochloride, phenazopyridine‐d5, acetaminophen, acetaminophen‐d4, and aniline were used; phenazopyridine, phenazopyridine‐d5, acetaminophen, and acetaminophen‐d4 were made in DMASO, while aniline was made in methanol. Interday accuracy and precision data is shown in Table S1.

Plasma samples were prepared by combining 100 μL of plasma with 400 μL of the internal standard addition solution in 2 mL polypropylene (PP) microcentrifuge tubes. The protein precipitated samples were briefly shaken and then placed on a vortex table to extract for 5 min before being briefly centrifuged (Eppendorf Microcentrifuge Model 5415R) at 13 200 RPM. The resulting supernatant solution (100 μL) was combined with 100 μL of ddH2O in a 2 mL amber autosampler vials with glass low volume inserts and briefly vortexed before being placed in the refrigerated autosampler of the UPLC–MS/MS for analysis.

Urine samples were prepared as follows. 30 μL of 0.95 M Na_2_HPO_4_ + 0.5% ascorbic acid in H_2_O were added to a 2 mL microcentrifuge tube, then 1.4 mL of EtOAc + 0.1% pyrogallol were added. 10 μL internal standard solution were added, followed by μL urine sample. The tube was briefly shaken and vortexed to mix, then extracted on a vortex table for 10 min, followed by centrifugation at 13 200 RPM to separate the layers. The EtOAc layer extract was then transferred to fresh 4 mL amber vials and dried down under nitrogen at room temperature for approximately 10 min. The dried sample was reconstituted in 0.5 mL of M50/50 + 0.01% AA. The sample was again vortexed for 2 min, then the extract (250 μL) transferred to a 2 mL amber vial with glass low volume insert and placed in the refrigerated autosampler of the UPLC–MS/MS for analysis.

Sample extracts were subjected to chromatographic separation performed on a waters H‐class UPLC system with an BEH amide HILIC column (waters acquity UPLC BEH amide, 100 mm length × 2.1 mm ID × 1.8 μm) and matching guard column (waters acquity UPLC BEH amide VanGuard pre‐column, 5 mm length × 2.1 mm ID × 1.8 μm) maintained at 50°C. Five microliters of sample were injected onto the column using a refrigerated autosampler maintained at 6°C. Mobile phase A consisted of 0.05% heptafluorobutyric acid (HFBA) in water, mobile phase B consisted of 0.05% HFBA in methanol. The mobile phase was delivered to the UPLC column at a flow rate of 0.3–0.5 mL per min. This method was modified from the plasma method but validated in the same fashion. Two resources were consulted for urine phenazopyridine analysis [25, 26].

A five‐point calibration curve made up in blank plasma and urine was prepared in the same manner as the samples but was spiked with a range of approximately 10–1000 ng/mL plasma and 10–2000 ng/mL urine for each analyte. Using these standards, calibration curves were constructed for each of the individual components using the MassLynx software (V4.2, Waters Corporation, Milford MA, USA) to determine analyte concentration in samples based on the sample/IS ratio. The limit of quantification (LOQ) was 10 ng/mL for all analytes in plasma and urine, as determined by the lowest concentration on the calibration curve. The limit of detection (LOD) was calculated as a signal to noise ratio (S/N) equal to 3. In plasma, this was 0.5 ng/mL for phenazopyridine and 1.5 ng/mL for acetaminophen and aniline. In urine, the LOD was 0.25 ng/mL for phenazopyridine, 5 ng/mL for acetaminophen, and 1 ng/mL for aniline.

### 2.7 Pharmacokinetic Parameter Determination and Statistical Analysis

Phoenix (Phoenix WinNonlin, version 8.0, Certara, St. Louis, MO) was used to determine the derived pharmacokinetic parameters. Plasma and urine concentrations were plotted on linear and semi‐logarithmic graphs to assess which model would fit best for the pharmacokinetic analysis. A non‐compartmental model was determined to be the most appropriate based upon visual inspection for goodness of fit and residual plots. The elimination half‐life (*T*_1/2_), maximum plasma concentration (*C*_max_), area under the curve (AUC_all_), area under the first moment curve (AUMC) and mean residence time (MRT_last_) were calculated. Individual values as well as geometric means for each parameter were determined.

## 3 Results

### 3.1 Study Cohort

Six goats with obstructive urolithiasis undergoing tube cystostomy surgery were enrolled in this study from August 2022 to March 2023. The mean goat age was 2.5 years (range 3 months to 4 years) and weight was 41.0 kg (range 9.3–59.4 kg). All goats were males, and 5/6 were castrated before presentation. Calcium carbonate calculi were ultimately diagnosed in 4/6 goats, magnesium calcium phosphate carbonate in 1 goat, and a mixed urolith opulation in 1 goat (20% magnesium ammonium phosphate and 80% magnesium calcium phosphate carbonate). Urolith type, as well as clinicopathologic data and ancillary treatments for the individual goats can be seen in Table S2.

### 3.2 Plasma and Urine Phenazopyridine Pharmacokinetics

For phenazopyridine assay validation, plasma had an *R* ^2^ > 0.99 with an interday CV% < 4 and an intraday CV% < 6. For urine, the assay had an *R* ^2^ > 0.98, an interday CV% < 12 and an intraday CV% < 6.

The pharmacokinetic parameters for both plasma and urine samples were determined using a non‐compartmental analysis. Individual and mean pharmacokinetic parameters are summarized in Table S3 Harmonic mean plasma elimination half‐life (*T*_1/2_) was 0.55 h (range 0.22–1.57 h), and the geometric mean maximum plasma concentration (*C*_max_) was 263.4 ng/mL (range 137.35–1047.88 ng/mL). The geometric mean time to maximum concentration (*T*_max_) was 0.35 h (range 0.25–0.5 h). The geometric mean area under the curve (0 to 12 h) was 221.61 h*ng/mL (range 52.55–1017.07 ng/mL). The geometric mean for AUMC was 172.87 h*h*ng/mL (range 16.39–896.51) and the geometric mean for the MRT was 0.78 (range 0.31–1.92).

**FIGURE 2:**
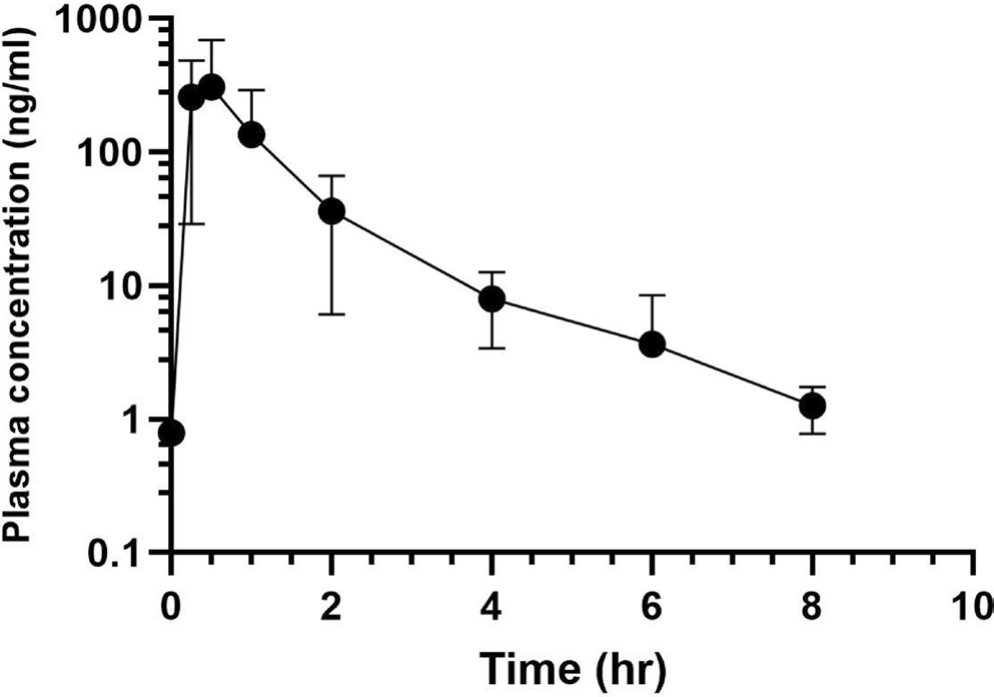
Plot of mean plasma phenazopyridine concentration after administration of first dose of oral phenazopyridine (*n* = 6 goats, 4 mg/kg; LOQ = 10 ng/mL; LOD = 0.5 ng/mL).

Mean plasma concentrations after the first dose of phenazopyridine are presented in Figure 2. As demonstrated in Table S3, the time to maximum concentration is short. At 6 h, the plasma concentration of phenazopyridine fell below the limit of detection/limit of quantification.

Of all urine samples collected (*n* = 52), only one sample had phenazopyridine concentrations above the assay limit of quantification (LOQ 10 ng/mL; phenazopyridine concentration 36.9 ng/mL). Therefore, pharmacokinetic analysis of phenazopyridine in urine was not performed.

### 3.3 Plasma and Urine Phenazopyridine Metabolite Pharmacokinetics

**FIGURE 3:**
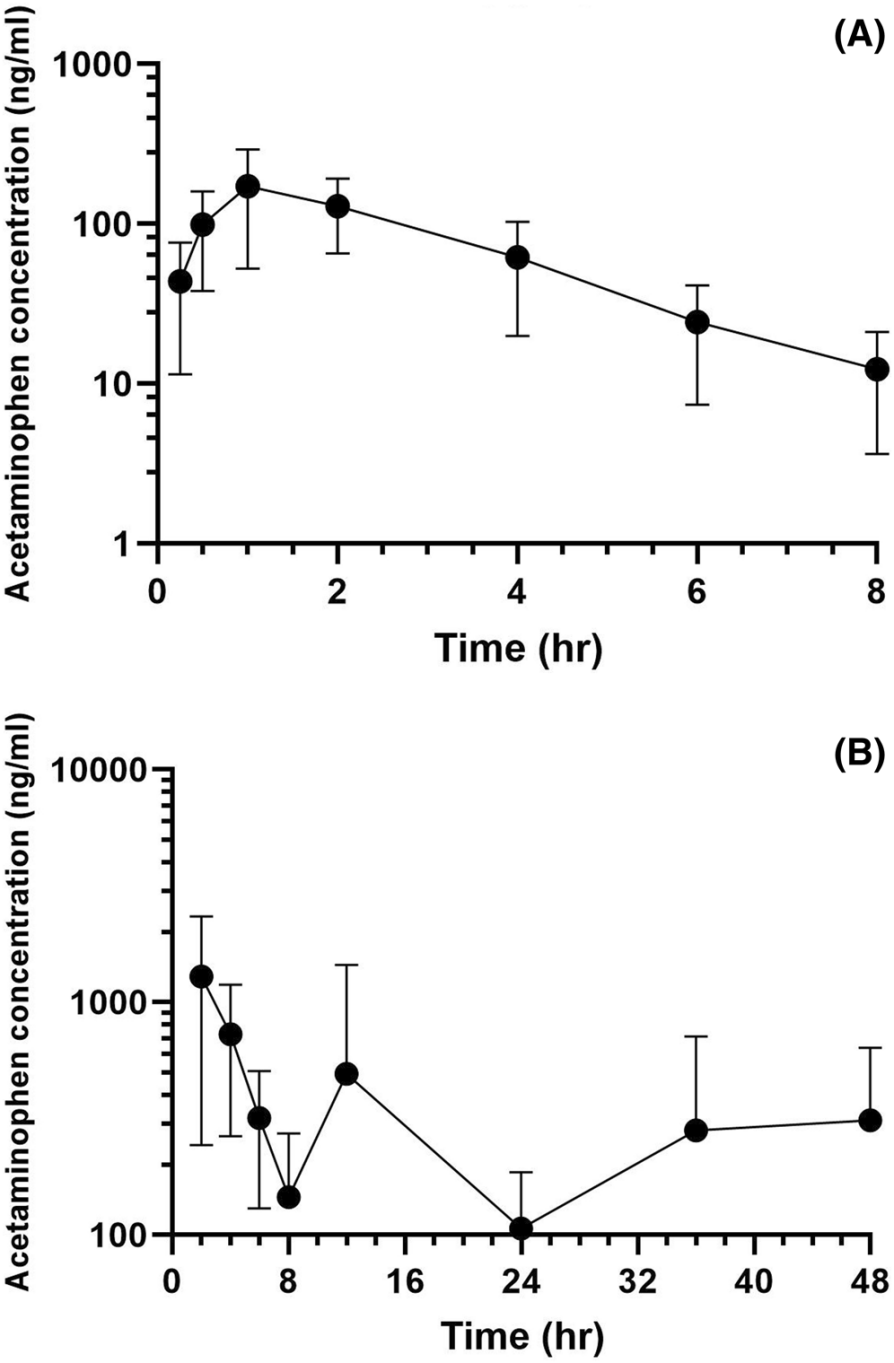
(A) Plot of mean plasma acetaminophen concentration after administration of first dose of oral phenazopyridine (*n* = 6 goats, 4 mg/kg; LOQ = 10 ng/mL; LOD = 1.5 ng/mL). (B) Mean urine acetaminophen concentration throughout the study period (*n* = 6 goats, LOQ = 10 ng/mL; LOD =5 ng/mL).

**FIGURE 4:**
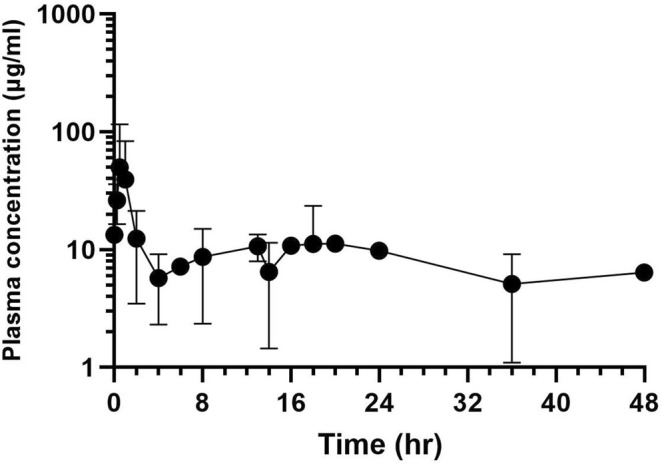
Plot of mean plasma aniline concentration after administration of first dose of oral phenazopyridine (*n* = 6 goats, 4 mg/kg; LOQ = 10 ng/mL; LOD = 1.5 ng/mL).

Acetaminophen and aniline were the primary metabolites studied. The LOQ and LOD for plasma and urine acetaminophen concentrations were 10; 1.5 and 10; 5 ng/mL, respectively. For aniline, the LOQ and LOD for plasma were 10 and 1.5 ng/mL. Figure 3 demonstrates the mean plasma acetaminophen concentrations (3A) and mean urine acetaminophen concentrations (3B). In the plasma, the maximum concentration of acetaminophen was 159.8 ng/mL with a time to maximum concentration of 1.2 h. The maximum acetaminophen concentration in urine was 3079 ng/mL, which was reached approximately 2 h after the initial dose. Aniline, a known toxic metabolite of phenazopyridine, was identified in plasma (Figure 4). The maximum plasma aniline concentration was 181.88 ng/mL and the time to maximum concentration was 0.5 h.

## 4 Discussion

This study reports the pharmacokinetics of orally administered phenazopyridine in goats. In this study, six male goats were hospitalized for urolithiasis and underwent a tube cystostomy procedure. They were enrolled and administered 4 mg/kg of phenazopyridine orally. With this dose, the harmonic mean phenazopyridine half‐life was determined to be 0.5 h, with a geometric mean maximum plasma concentration of 263.4 ng/mL and geometric mean time to maximum concentration of 0.35 h. There was only one urine sample with detectable phenazopyridine. The two metabolites assessed were acetaminophen and aniline and relatively low levels of both were found in the plasma.

The true underlying mechanism of action of phenazopyridine has not been clearly elucidated. Historically, phenazopyridine was thought to have antimicrobial properties and was administered in humans for the treatment of urinary tract infections [10]. This was quickly debunked, but phenazopyridine was still used as an ancillary therapy for urinary tract infections as it mitigated pain associated with urination [10]. In rats, increased doses of phenazopyridine inhibit Aδ nerve fibers [15]. In this rat study, phenazopyridine was dosed intravenously; there is some suggestion that phenazopyridine can work topically, inhibiting voltage‐gated sodium channels like lidocaine; however, this has not been clearly demonstrated [15]. Phenazopyridine undergoes hepatic metabolism, with acetaminophen as one of the primary metabolites [10]. The role in which acetaminophen plays in mitigating pain as a metabolite to phenazopyridine is also not clearly understood. Acetaminophen might act locally in the urinary bladder, decreasing prostaglandin concentrations and allowing for increased comfort [15].

The authors were able to find four other studies assessing the pharmacokinetics of phenazopyridine in various species, different routes of administration, and different dosages [9, 11, 12, 13]. When comparing this study to the previous literature, goats administered oral phenazopyridine had a shorter half‐life when compared to humans (1.26–9 h [9, 13]); an increased maximum plasma concentration (in humans, 0.03 μg/mL) [11] and a shorter time to maximum concentration (in humans, 2 h) [9]. It is challenging to compare these studies as different doses and routes of administration were used. The goats in this study were receiving ancillary intravenous fluid therapy support to help correct the post renal azotemia; the increased intravenous fluid therapy might have contributed to increased diuresis and a shortened half‐life when compared to the human population (Table S2). However, we would have expected the plasma phenazopyridine concentration to be more like the reported human and rat plasma concentration. A potential reason for the increased plasma phenazopyridine concentration for the goats in this study could relate to any renal impairment secondary to urinary obstruction. With current diagnostics, it is challenging to understand if there is any true intrinsic renal disease while goats display post renal azotemia.

Although not demonstrated, there was increased variability of plasma phenazopyridine concentrations. In ruminants, the rumen plays a huge role with absorption, distribution, and excretion of orally administered medications. Differences in rumen size, volume, and microbiota communities can all impact how readily absorption will occur. Additionally, the rumen pH might affect the solubility of phenazopyridine and therefore, its rate of gastrointestinal absorption. It has been demonstrated that in an environment with a pH of 6, there is decreased solubility of phenazopyridine, allowing for a short time for maximum concentration to be reached [27]. The normal rumen pH in healthy goats is approximately 6–7, depending on factors such as grain and forage intake. This might have allowed for increased phenazopyridine absorption compared to humans. It is important to note that the rumen pH was not obtained for goats enrolled in this study; with their presenting clinical signs, however, it is likely they did not have appropriate rumen contractions and might have been anorexic leading up to presentation, causing an altered rumen environment. This could have affected consequent absorption and plasma concentrations observed.

In the current study, phenazopyridine was not detected in the urine. This was unusual, as phenazopyridine is labeled to mitigate pain associated with dysuria in humans. Phenazopyridine is eliminated through the urinary tract. In this study, the goats received ancillary fluid therapy. The use of intravenous fluid therapy is thought to contribute to augmented renal clearance, which is when there is increased elimination of circulating solutes compared to normal [28]. The use of intravenous fluid therapy during and after surgery for these enrolled goats could have contributed to enhanced phenazopyridine urine clearance, resulting in diluted urine and an inability to detect phenazopyridine. In this study, four out of the six goats presented with an azotemia (Table S2). This azotemia is likely pre‐renal in nature; however, there might have been a renal component, impairing the excretion of phenazopyridine. Unfortunately, it is challenging to discern, before surgery, whether the azotemia is pre‐renal or renal in nature. It is important to note that, with oral phenazopyridine administration, the urine becomes pigmented in humans and horses [29]. For the goats enrolled in this study, their urine did not become pigmented, perhaps secondary to intravenous fluid therapy and augmented renal clearance.

The two metabolites studied were acetaminophen and aniline. The role of acetaminophen in mitigating pain associated with dysuria is not completely known. In humans, the effective serum acetaminophen concentration that alleviates fevers 50% of the time is estimated to be between 15.2 and 16.5 μg/mL [30]. Both the plasma and urine acetaminophen concentrations were well below the estimated effective range in humans. With the low plasma and urine concentrations, we question acetaminophen's role in pain mitigation.

Aniline is a toxic compound that is irritating to the skin, eyes, and respiratory tract [31]. Aniline induces methemoglobinemia by converting Fe^2+^(iron) to Fe^3+^ in hemoglobin, impairing oxygen delivery to tissues. In humans, aniline toxicity is typically a result of inhalation, but as little as 1 g ingested can be fatal to humans [31]. In this study, the aniline plasma concentrations in goats were quite low, indicating the risk of adverse effects associated with methemoglobinemia development might also be low. Increased dosages or frequency of phenazopyridine administration, or both, might thus be possible, though a safety or toxicity study would be necessary before clinical use of such treatment regimens. It should be noted that some veterinary organizations, such as the American College of Veterinary Pharmacists, state that phenazopyridine should not be used in animals; there might be potential risks associated with using phenazopyridine; however, no adverse reactions were noted in this study. Phenazopyridine has the potential to be safely used in goats, or the ancillary therapies provided, such as intravenous fluid therapy, helped reduce any adverse effects.

There were several limitations in this study. All goats in this study were orally drenched with phenazopyridine dissolved in water. It is possible that crushing and dissolving for the phenazopyridine tablet(s) might have impacted phenazopyridine absorption. Additionally, while no study personnel reported incomplete administration of each goat's complete phenazopyridine dose, it is possible that partial doses were lost in oral administration, impacting the ultimate dose administered. Importantly, this pharmacokinetic trial was conducted in goats with clinical disease. It is challenging to understand baseline pharmacokinetic principles with present co‐morbidities, ancillary treatments and individual goat variability. Moving forward, a pharmacokinetic study performed in healthy goats would be critical to understand an effective dosing regimen, which then could be assessed in a clinical diseased state. In particular, intravenous fluid therapy administration in addition to changes in renal function might have altered clearance and accurate urine phenazopyridine concentrations. Nevertheless, this study cohort represents the group of goats in which phenazopyridine is most commonly utilized, making the findings of this study clinically informative.

There is no FDA approved label for the use of phenazopyridine as a urinary bladder analgesic in ruminants, or any food producing species. The use of phenazopyridine in ruminants would be considered an extra label drug use, in which the Food Animal Residue Avoidance Databank (FARAD) would need to be contacted for an extended meat or milk withdrawal interval. This clinical study would be one of the first phenazopyridine pharmacokinetic trials conducted in ruminants; therefore, FARAD would provide conservative meat and milk withdrawal intervals using data extrapolated from other species.

Currently, the use of phenazopyridine orally at a dose of 4 mg/kg every 12 h appears to be well tolerated in goats, but the pharmacokinetics in clinically affected goats reveal that phenazopyridine might not concentrate at clinically relevant concentrations within the urogenital tract.

## Disclosure

Both the indication and route for ceftiofur sodium are off‐label uses of antimicrobials as described in Table S2.

## Ethics Statement

This work was approved by the North Carolina State University's Institutional Animal Care and Use Committee (PROTOCOL 22‐295). Authors declare human ethics approval was not needed.

## Conflicts of Interest

The authors declare no conflicts of interest.

## Supporting information


**Table S1.** Interday accuracy and precision data for phenazopyridine and its metabolites in goat plasma and urine. Quality control samples were used at low, medium, and high concentrations based on the calibration curve (10, 100, 1000 ng/mL, respectively). All parameters were calculated using five replicates of each analyte, with the exception of aniline, which was calculated using only three replicates.

**Table S2.** Urolith type, clinicopathologic data and ancillary treatments for the individual goats.

**Table S3.** Individual and geometric/harmonic mean + geometric CV%/harmonic standard deviation for plasma pharmacokinetic parameters after first dose of oral phenazopyridine (4 mg/kg). Abbreviations: AUC0–12 h, area under the curve for time period 0–12 h; AUMC, area under the moment curve; *C*_max_, maximum concentration; MRT, mean residence time; *T*_1/2_, elimination half‐life; *T*_max_, time to maximum concentration.
