# Supplementary material for: Pharmacokinetics of Orally Administered Phenazopyridine in Goats With Obstructive Urolithiasis
Source: J Vet Intern Med. 2025 Jun 26;39(4):e70167. doi: 10.1111/jvim.70167 (PMC12199991; doi:10.1111/jvim.70167)
Supplement: Supplementary file 1 — Table S1. Interday accuracy and precision data for phenazopyridine and its metabolites in goat plasma and urine. Quality control samples were used at low, medium, and high concentrations based on the calibration curve (10, 100, 1000 ng/mL, respectively). All parameters were calculated using five replicates of each analyte, with the exception of aniline, which was calculated using only three replicates. [file JVIM-39-e70167-s001.docx]

Supplemental Table 1. Interday accuracy and precision data for phenazopyridine and its metabolites in goat plasma and urine. Quality control samples were used at low, medium, and high concentrations based on the calibration curve (10, 100, 1000 ng/mL, respectively). All parameters were calculated using 5 replicates of each analyte, with the exception of aniline, which was calculated using only 3 replicates.

|  | **Phenazopyridine** | | **Acetaminophen** | | **Aniline** | |
| --- | --- | --- | --- | --- | --- | --- |
| **Plasma** | | | | | | |
|  | *Accuracy* | *Precision* | *Accuracy* | *Precision* | *Accuracy* | *Precision* |
| 10 ng/mL | 102% | 3.84% | 104% | 4.81% | 94.0% | 13.7% |
| 100 ng/mL | 102% | 3.51% | 101% | 4.78% | 104% | 4.51% |
| 1000 ng/mL | 101% | 2.86% | 99.9% | 4.26% | 101% | 5.87% |
| **Urine** | | | | | | |
|  | *Accuracy* | *Precision* | *Accuracy* | *Precision* | *Accuracy* | *Precision* |
| 10 ng/mL | 105% | 10.1% | 102% | 11.5% | 108% | 5.81% |
| 100 ng/mL | 97.1% | 5.0% | 95.7% | 3.21% | 101% | 3.90% |
| 1000 ng/mL | 102% | 4.82% | 101% | 1.79% | 102% | 2.96% |
