# Supplementary material for: Pharmacokinetics of Orally Administered Phenazopyridine in Goats With Obstructive Urolithiasis
Source: J Vet Intern Med. 2025 Jun 26;39(4):e70167. doi: 10.1111/jvim.70167 (PMC12199991; doi:10.1111/jvim.70167)
Supplement: Supplementary file 3 — Table S3. Individual and geometric/harmonic mean + geometric CV%/harmonic standard deviation for plasma pharmacokinetic parameters after first dose of oral phenazopyridine (4 mg/kg). Abbreviations: AUC0–12 h, area under the curve for time period 0–12 h; AUMC, area under the moment curve; C max, maximum concentration; MRT, mean residence time; T 1/2, elimination half‐life; T max, time to maximum concentration. [file JVIM-39-e70167-s003.docx]

Supplemental Table 3. Individual and geometric/harmonic mean + Geometric

CV%/harmonic standard deviation for plasma pharmacokinetic parameters after first

dose of oral phenazopyridine (4 mg/kg). Abbreviations: T1/2: Elimination Half-life; Cmax:

Maximum concentration; Tmax: Time to maximum concentration; AUC0-12hr: Area under

the curve for time period 0-12 hour; MRT: mean residence time; AUMC: area under the

moment curve.

| **Animal** | **T_1/2_ (hours)** | **C_max_ (ng/mL)** | **T_max_ (hours)** | **AUC_0-12hr_**  **(hr*ng/mL)** | **AUMC (hr*hr*ng/mL)** | **MRT (hr)** |
| --- | --- | --- | --- | --- | --- | --- |
| Goat 1 | 1.35 | 190.12 | 0.25 | 150.83 | 110.07 | 0.73 |
| Goat 2 | 0.22 | 178.92 | 0.25 | 52.55 | 16.39 | 0.31 |
| Goat 3 | 0.29 | 189.5 | 0.25 | 126.6 | 71.43 | 0.56 |
| Goat 4 | 1.2 | 137.35 | 0.5 | 270.86 | 518.92 | 1.92 |
| Goat 5 | 1.57 | 359.95 | 0.5 | 428.53 | 445.13 | 1.04 |
| Goat 6 | 1.57 | 1047.88 | 0.5 | 1017.07 | 896.51 | 0.88 |
| *Mean (Geometric)* | 0.55 *(Harmonic mean*) | 263.4 | 0.35 | 221.61 | 172.9 | 0.78 |
| *Geometric CV%* | 1.59 (*Harmonic Standard Deviation)* | 86.49 | 39.38 | 137.97 | 294.5 | 67 |
